# A benchmark data set for the mechanical properties of double-stranded DNA and RNA under torsional constraint

**DOI:** 10.1016/j.dib.2020.105404

**Published:** 2020-03-12

**Authors:** Willem Vanderlinden, Pauline J. Kolbeck, Franziska Kriegel, Philipp U. Walker, Jan Lipfert

**Affiliations:** Department of Physics and Center for Nanoscience, LMU Munich, Amalienstrasse 54, 80799 Munich, Germany

**Keywords:** DNA, RNA, Torsional stiffness, Linking number, Magnetic tweezers, Single-molecule, Mechanics

## Abstract

Nucleic acids are central to the storage and transmission of genetic information and play essential roles in many cellular processes. Quantitative understanding and modeling of their functions and properties requires quantitative experimental characterization. We use magnetic tweezers (MT) to apply precisely calibrated stretching forces and linking number changes to DNA and RNA molecules tethered between a surface and superparamagnetic beads. Magnetic torque tweezers (MTT) allow to control the linking number of double-stranded DNA or RNA tethers, while directly measuring molecular torque by monitoring changes in the equilibrium rotation angle upon over- or underwinding of the helical molecules. Here, we provide a comprehensive data set of double-stranded DNA and RNA under controlled stretching as a function of the linking number. We present data for extension and torque as a function of linking number in equilibrium. We report data for the critical torque of buckling and of the torsional stiffness of DNA and RNA as a function of applied force. Finally, we provide dynamic data for the hopping behavior at the DNA buckling point.

specifications table

**Table:** 

Subject	Biophysics
Specific subject area	Single-molecule magnetic tweezers measurements of double-stranded DNA and RNA under torsional constraint
Type of data	Graph Figure
How data were acquired	Data were acquired using custom-built magnetic tweezers instruments. Data acquisition used LabView software and post-processing was carried out using Matlab-based routines.
Data format	Raw Analyzed
Parameters for data collection	Data were collected under near-physiological ionic strength (100–150 mM monovalent salt), unless otherwise indicated.
Description of data collection	We used multiple biotin-streptavidin and dioxigenin-antidig bonds to tether linear double stranded nucleic acids between the bottom surface of a flow cell and paramagnetic beads, such that they are torsionally constrained. A home-build magnetic tweezers setup equipped with external magnets, arranged either in magnetic torque tweezer or in (conventional) vertical anti-parallel geometry, was employed to measure the torque accumulation and changes in tether extension in response to magnet rotation.
Data source location	Delft, The Netherlands and Munich, Germany
Data accessibility	Repository name: Mendeley Data Data identification number: doi:10.17632/3228jznyct.1 Direct URL to data: http://dx.doi.org/10.17632/3228jznyct.1
Related research article	Data shown are from several articles, cited in the corresponding sections. The main reference is Lipfert J, Skinner GM, Keegstra JM, Hensgens T, Jager T, Dulin D, Köber M, Yu Z, Donkers SP, Chou FC, Das R, Dekker NH Double-stranded RNA under force and torque: Similarities to and striking differences from double-stranded DNA. Proc Natl Acad Sci U S A, 111, 15,408-15,413 (2014) DOI: 10.1073/pnas.1407197111

## Value of the data

• Nucleic acids are central to the storage and processing of nucleic acids and increasingly used in nanotechnology. The mechanical properties of DNA and RNA fundamentally enable and constrain their biological functions and technological uses and, therefore, high-quality data are needed to build a quantitative understanding and optimize their uses.
  We expect our data to be beneficial to experimentalist with assays that involve double-stranded DNA and RNA, in particular at the single-molecule level and under torsional constraint. In addition, they serve as a benchmark for researchers in the field of biomolecular simulations, to test and improve simulations.
• DNA and RNA held under torsional constraint in magnetic tweezers are frequently used in single-molecule assays that probe nucleic acids processing enzymes, such as polymerases, helicases, topoisomerases, and recombinases. A quantitative understanding of the mechanics and dynamics of nucleic acids in magnetic tweezers is a pre-requisite to interpreting and improving such single-molecule experiments.
• There are a number of approaches to modeling DNA and RNA, ranging from coarse grained simulations to all atom or even quantum mechanical treatment. All these models feature free or adjustable parameters that need to be optimized against experimental data.

## 1 Data

**Fig. 1:**
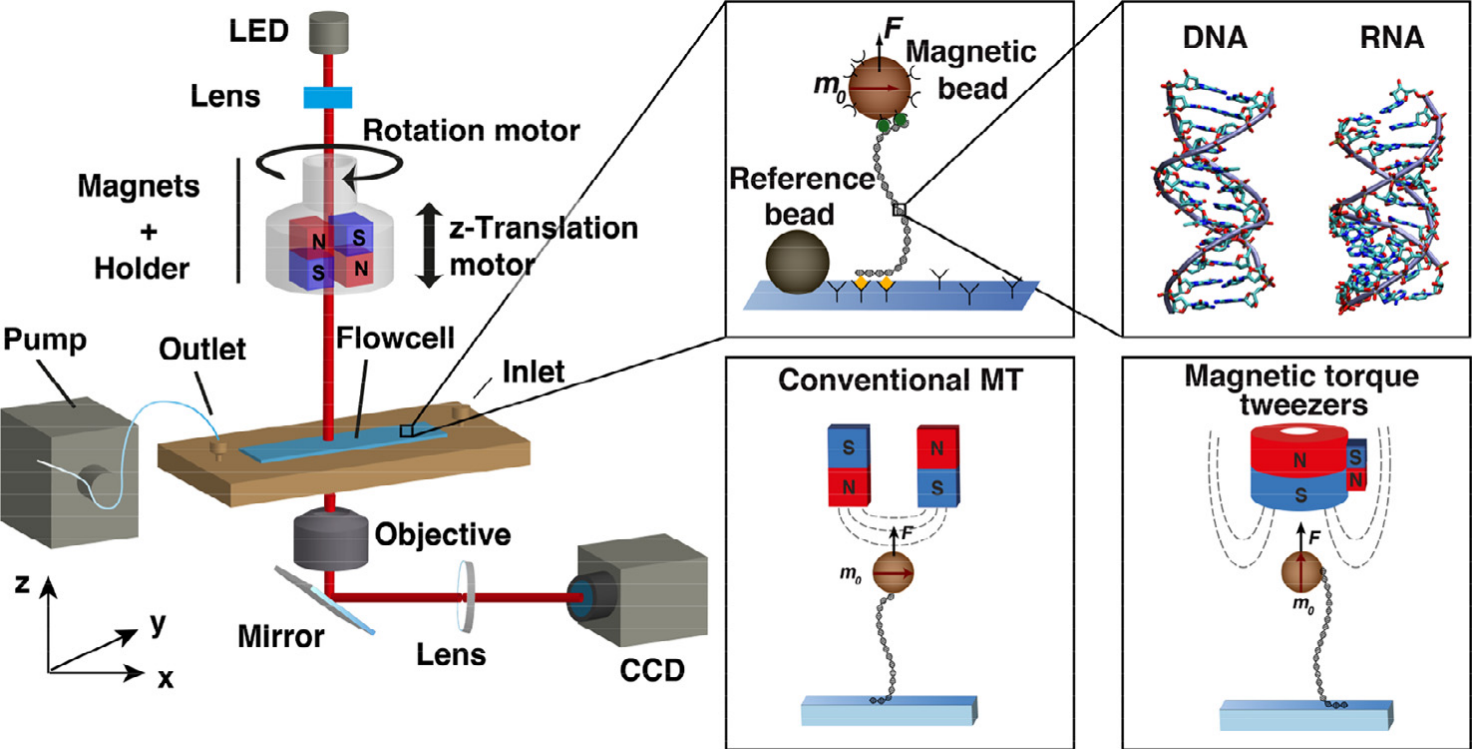
Magnetic tweezers setup for measuring extension and torque of double stranded nucleic acids. A flow cell build from two cover slips interspaced by a layer of parafilm pre-cut to form a flow channel is mounted in a holder that enables liquid exchange using a peristaltic pump. Permanent magnets are mounted on a rotational motor stage and on a z-translation stage, such that both linking number and applied force in the nucleic acid tether can be changed. Monochromatic light from an LED illuminates the flow cell and the micron-sized beads through a gap in the magnet assembly. An oil-immersion objective mounted on a piezo-stage enables positioning of the focal plane, and guides the light via a mirror and a tube lens to a camera that is connected to a computer equipped with software for data read-out and bead tracking. *Inset middle top*: Nucleic acid molecules are tethered via multiple non-covalent interaction to the bottom slide of the flow cell at one end, and to streptavidin-coated paramagnetic beads at the other. Non-magnetic, polystyrene beads are stably bound to the bottom glass coverslip of the flow cell and serve as fiducial markers for drift correction. *Inset right top:* Molecular structures of double-stranded DNA and RNA, rendered from the PDB structures with accession codes 2BNA and 1RNA, respectively. *Insets bottom:* In conventional magnetic tweezers the external magnets are arranged in a horizontal anti-parallel geometry. In magnetic torque tweezers, a cylindrical magnet with smaller side magnet is used to apply a predominantly vertical field, to reduce the torsional trap stiffness.

We present data on the response of double-stranded DNA and double-stranded RNA that are over- and underwound while being held under constant stretching forces. All data were recorded using custom-built magnetic tweezers (MT) instruments (Fig. 1). In MT, molecules are tethered between a flow cell surface and magnetic beads [1,2]. We used a 7.9 kbp DNA construct [3], [4], [5] and a 4.2 kbp RNA construct [6] that were attached to the surface and bead by multiple digoxigenin and biotin labels (Fig. 1**,** *inset middle top*), respectively. In the case of DNA, biotin and digoxigenin labeled fragments were generated separately in PCR reactions and ligated to the central, unmodified, DNA segment. For RNA, multiple biotin and streptavidin labels were incorporated using a two-step T7 RNA polymerase in vitro transcription protocol [6] (see *Experimental Design, Materials, and Methods* for details).

**Fig. 2:**
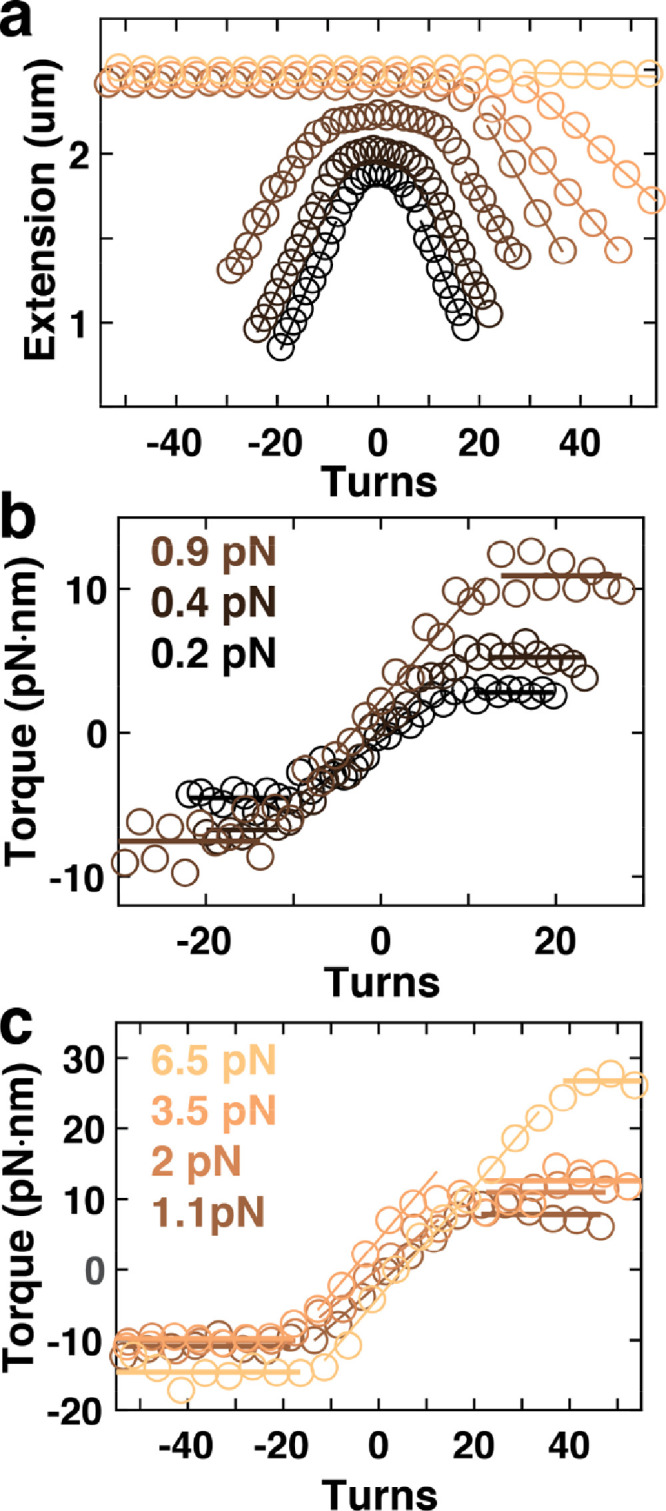
Extension-rotation and torque-rotation curves for double-stranded DNA. (**a)** Extension vs. applied turns curves for 7.9 kbp dsRNA at F = 0.2, 0.4, 0.9, 1.1, 2, 3.5, and 6.5 pN (dark to light). Solid lines indicate linear fits in the plectonemic regime for forces < 6 pN and for the B- to P-DNA transition regime for the highest force shown. (**b)** and (**c)** Torque vs. applied turns curves corresponding to the measurements in panel a. Horizontal lines are fits to the torque plateaus to determine buckling and melting torques. Sloped lines are linear fits to determine the torsional stiffness.

**Fig. 3:**
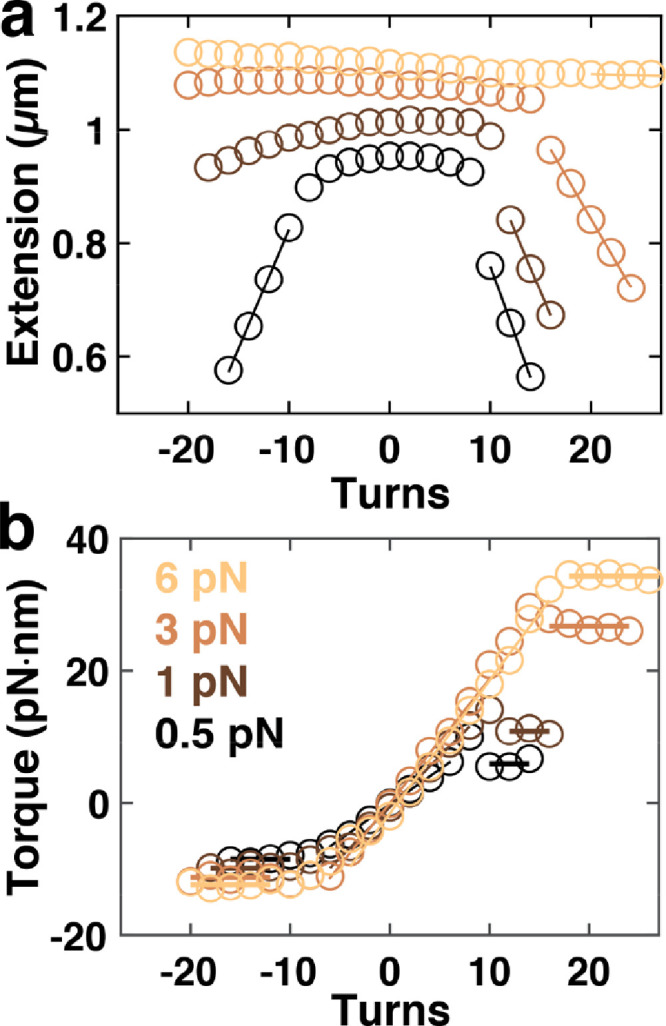
Extension-rotation and torque-rotation curves for double-stranded RNA. a) Extension vs. applied turns curves for 4.2 kbp dsRNA at F = 0.5, 1, 3, and 6.5 pN (dark to light). Solid lines indicate linear fits in the plectonemic regime for forces < 6 pN and for the B- to P-RNA transition regime for the highest force shown. b) Torque vs. applied turns curves corresponding to the measurements in panel a. Horizontal lines are fits to the torque plateaus to determine buckling and melting torques. Sloped lines are linear fits to determine the torsional stiffness.

For both types of molecules, we performed experiments where the molecules were systematically over- and underwound by a certain number of turns by rotating the magnets (Figs. 2 and 3). The number of applied turns corresponds to the change in linking number *ΔLk*, where zero turns corresponds to a torsionally relaxed molecule. Bead positions were tracked in real time [7] in (x,y,z), which enabled determination of the DNA or RNA tether extension (simply from the z-position above the surface) and of the rotation angle *θ* via analysis [4,^6^,8] of the position in (x,y) (see “*Experimental Design, Materials, and Methods*”). At each number of applied turns, (x,y,z)-traces of 200 s were recorded. The corresponding average extensions are shown as a function of the applied turns (Figs. 2a and 3a). The rotation angle determined from the (x,y)-positions are converted to torque Γ using the relationship [3,^9^,10]

(1) $$\Gamma =\;-\frac{k_{B}T}{Var\left(\theta \right)}\langle \theta_{N}-\theta_{0}\rangle$$

where *k_B_* is the Boltzmann constant, *T* the temperature, Var(…) denotes the variance and <…> the average, both taken over the 200 s time traces, and *θ_N_* and *θ_0_* the rotation angle after *N* and zero turns, respectively. Torques are similarly shown as a function of applied turns (Figs. 2b,c and 3b). The plateaus in the torque data at positive turns correspond to the critical torque for buckling or buckling torque for short (solid lines at positive turns in Figs. 2b,c and 3b). Corresponding plateaus at negative turns correspond to the buckling torque for forces < 1 pN and to the critical torques for melting (i.e., torque-induced opening of base pairs) [11] for forces > 1 pN. Finally, the parts around zero turns in the torque vs. turns relations are well described by linear fits (linear slopes in Figs. 2b,c and 3b). The fitted slopes *ΔΓ*/*ΔN* are related to the torsional stiffness *C* (i.e., the torsional persistence length in nm) of the molecules via

(2) $$C=\;\frac{L_{c}}{2\pi k_{B}T}\frac{\Delta \Gamma }{\Delta N}$$

where *L_C_* is the contour length of the DNA or RNA molecule. All data contained in Figs. 2 and 3 are saved in text files called “DNA_Extension_vs_Turns_XXpN.txt”, “DNA_Torque_vs_Turns_XXpN.txt”, “RNA_Extension_vs_Turns_XXpN.txt”, and “RNA_Torque_vs_Turns_XXpN.txt”, where XX is the corresponding force. In the text files, the first column denotes the number of applied turns (= *ΔLk*) and the second column the corresponding mean extension (in µm) or torque (in pN⋅nm), respectively.

## 2 Buckling torques and torsional persistence length

**Fig. 4:**
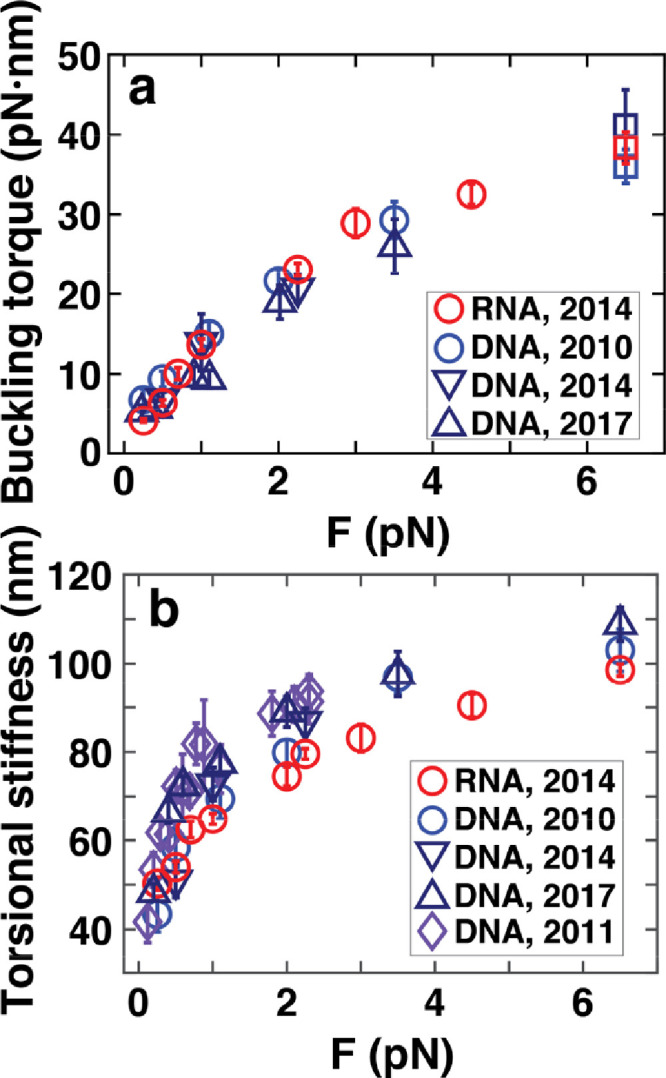
Buckling torques and torsional persistence lengths for double-stranded DNA and RNA. **(a)** Critical torques for buckling for overwound double-stranded DNA [3,^4^,6] and RNA [6] molecules in near-physiological ionic strength (100–150 mM monovalent ions). The points at 6.5 pN do not correspond to buckling, but to the B-to-P DNA and A-to-P RNA transitions [6,[21], [22], [23]. **(b)** Effective torsional persistence length as a function of applied stretching force for double-stranded DNA (3,4,6,12) and RNA [6]. (For interpretation of the references to color in this figure, the reader is referred to the web version of this article.)

Using torque vs. rotation measurements similar to the ones shown in Figs. 2 and 3, we have systematically determined the buckling torques and torsional stiffnesses of double-stranded DNA and RNA using Eq. 2. Here, we present a comprehensive data set of buckling torques (Fig. 4a) and torsional persistence lengths *C* (Fig. 4b) for several DNA constructs and for double-stranded RNA. All data were obtained in 100–150 mM monovalent salt, corresponding to approximately physiological ionic strength. The data set includes 4.2 kbp RNA measured in TE buffer (10 mM Tris–HCl, pH 8.0, and 1 mM EDTA) with 100 mM NaCl added [6] (Fig. 4a,b, red circles “RNA 2014”); 7.9 kbp DNA measured in PBS buffer [3] (10 mM phosphate buffer, pH 7.4, 137 mM NaCl and 3 mM KCl) (Fig. 4a,b, blue circles “DNA 2010”); 3.4 kbp DNA measured in TE buffer with 100 mM NaCl added [6] (Fig. 4a,b, downward pointing triangles “DNA 2014”); and 7.9 kbp DNA in TE buffer with 100 mM NaCl added [4] (Fig. 4a,b, upward pointing triangles “DNA 2017”). Data at stretching forces > 6 pN do not correspond to buckling but to structural transitions, namely the B-to-P transition for DNA and A-to-P transition for RNA [6], [21], [22], [23]. For comparison, we have included a data set for the torsional stiffness of DNA that was obtained by analyzing the rotational fluctuations of a DNA end that is held under constant stretching force, but allowed to rotate freely in so-called freely-orbiting magnetic tweezers [12] (FOMT; Fig. 4b, diamonds “DNA 2011”). In the FOMT, the torsional persistence is determined from the variance of the rotational (angle) fluctuations as *C* = *L_C_* / Var(*θ*). All data are provided in text files according to the table below. In all files the first column is force (in pN), second column buckling torque (in pN⋅nm) or torsional stiffness (in nm), respectively, and the third column the error on the buckling torque or torsional stiffness.

**Table:** 

Data set	File names	Reference
DNA2010	Lipfert_etal_2010_DNA_BucklingTorques.txt Lipfert_etal_2010_DNA_C_vs_F.txt	Ref. [3]
DNA2011	Lipfert_Wiggin_2011_FOMT_C_vs_F.txt	Ref. [12]
DNA2014	Lipfert_etal_2014_3p4kbpDNA_BucklingTorques.txt Lipfert_etal_2014_3p4kbpDNA_C_vs_F.txt	Ref. [6]
DNA2017	Kriegel_etal_2017_DNA_BucklingTorques.txt Kriegel_etal_2017_DNA_C_vs_F.txt	Ref. [4]
RNA2014	Lipfert_etal_2014_RNA_BucklingTorques.txt Lipfert_etal_2014_RNA_C_vs_F.txt	Ref. [6]

## 3 Dynamics of DNA supercoil nucleation

**Fig. 5:**
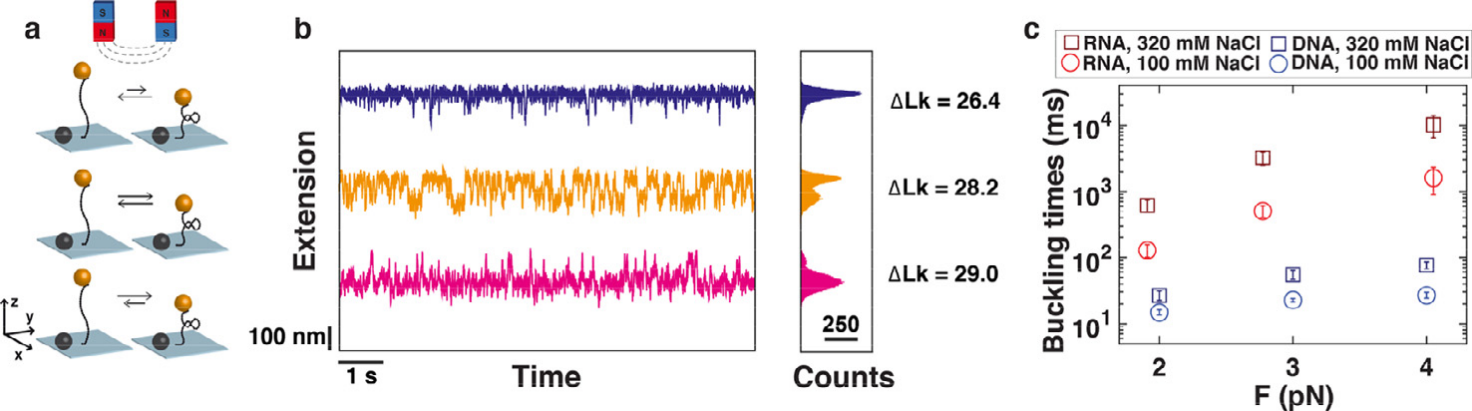
Quantifying the dynamics of supercoil nucleation using kHz bead tracking. (**a)** Schematic of the DNA or RNA buckling transition. (**b)** Extension vs. time traces at different values of ΔLk (the linking difference with respect to the relaxed state) for a 7.9 kbp DNA molecule in aqueous buffer supplemented with 100 mM NaCl. The molecule is attached to a 2.8 μm-sized magnetic bead, and subjected to F = 3 pN. (**c)** Characteristic buckling times, defined as the mean dwell time in the pre- and post-buckling state at the 50–50 point, i.e., at the linking number difference where the forward and backward rates for buckling are the same. Data for RNA [6] and DNA [15] in 100 mM and 320 mM NaCl.

Finally, we present data on the buckling dynamics [13,14]. The data were obtained with a 7.9 kbp DNA molecule tethered between the bottom of a flow cell surface at one end, and superparamagnetic beads at the other. Again, multiple attachment points at both ends assure torsionally constrained attachment, such that rotation of the external magnets changes the linking number *Lk* of the DNA. First, two calibration measurements are carried out. By measuring the bead extension versus the number of applied rotations at low force (*F* = 0.4 pN) we deduced the position of the rotation motor where a given DNA tether is torsionally relaxed, i.e., *Lk* = *Lk_0_*, under given conditions of ionic strength (see measurement file “FOV1_0_4pN_rot.txt” and corresponding motor file “FOV1_0_4pN_rot_motors.txt”). To deduce the full extension of the molecule, we record the z-position of the bead upon moving the z-translation motor from high force (5 pN) to low force (< 0.1 pN) and back (see measurement file “FOV1_length.txt” and corresponding motor file “FOV1_length_motors.txt”). Next, we record rotation curves at 1 kHz framerate at higher force within the regime of linking differences *ΔLk* = *Lk*-*Lk_0_* wherein plectonemic supercoils nucleate at force *F* = 3 pN and in a buffer with 100 mM NaCl. For accurate sampling of the supercoil nucleation, we program the rotation motor to make changes in steps of 0.2 turns (see Fig. 5; see measurement files

“FOV1_hopping_1kHz_3pN_M270_100mM_rotmotor28_0.txt”,
“FOV1_hopping_1kHz_3pN_M270_100mM_rotmotor28_2.txt”,
“FOV1_hopping_1kHz_3pN_M270_100mM_rotmotor28_4.txt”,
“FOV1_hopping_1kHz_3pN_M270_100mM_rotmotor28_6.txt”,
“FOV1_hopping_1kHz_3pN_M270_100mM_rotmotor28_8.txt”,
“FOV1_hopping_1kHz_3pN_M270_100mM_rotmotor29_0.txt”,
“FOV1_hopping_1kHz_3pN_M270_100mM_rotmotor29_2.txt”,
“FOV1_hopping_1kHz_3pN_M270_100mM_rotmotor29_4.txt”,
“FOV1_hopping_1kHz_3pN_M270_100mM_rotmotor29_6.txt”,
“FOV1_hopping_1kHz_3pN_M270_100mM_rotmotor29_8.txt”,
“FOV1_hopping_1kHz_3pN_M270_100mM_rotmotor30_0.txt”).

In the measurement files, the first column denotes the time in frames, the second column gives the elapsed time in ms, columns 3–5 indicate the x,y and z-positions of the reference bead (in µm), and columns 6–8 give the x,y, and z-positions of the tethered bead (also in µm).

We show three example traces around the buckling transition, where the molecule jumps between the pre- and post-buckling states (Fig. 5a,b). Dwell time analysis of the buckling times traces [14,15] yields the characteristic buckling time, which is the mean dwell time in the pre- and post-buckling state at the 50–50 point. Documented Matlab code that quantifies the thermodynamic and kinetic characteristics from the measurement data is provided as “Hopping_analysis_example.m”. The characteristic buckling times for double-stranded DNA [15] and RNA [6] in buffer with 100 mM NaCl and 320 mM NaCl are shown in Fig. 5c.

## 4 Experimental design, materials, and methods

### 4.1 Magnetic tweezers instruments

Measurements were performed using home-built MT setups [4], [5], [6]. We describe the configuration used for DNA measurements. The configuration used for RNA measurements is essentially the same, with some components from different vendors, with details provided in Ref. [6]. A light emitting diode (Osram Oslon SSL, red, 165 lm) was used to illuminate the sample from above. An oil immersion objective (60 × Plan Fluorite with correction collar, NA 0.9) was placed on a piezo (Pifoc, P-726.1CD and controller, E-753.1CD, Physik Instrumente) stage to adjust the focus position. Beads were imaged with a mirror (20D20ER.1, Newport) and tube lens (G322304000, Newport) onto the chip of a camera (Falcon PT-41-4M60, Dalsa). Magnets (see below) were placed on a motorized arm, using a translational motor (C-863.11-Mercury controller and M-126.PD2 motor, Physik Instrumente) and a rotational motor (C-863.11-Mercury controller and C- 150.PD motor, Physik Instrumente) to control the magnets’ rotation and height above the flow cell. DNA or RNA tethers are in a flow cell that is connected to a pump (ISM832C, Ismatec) for buffer exchange. The setup is controlled using a computer (DELL Precision T3600) equipped with a framegrabber (PCIe-1433, National Instruments) and custom software [7] written in Labview (National Instruments).

### 4.2 DNA constructs

The DNA measurements shown in Figs. 2 and 5 used a 7.9-kbp DNA constructs [3] ligated at the ends to ~600-bp DNA PCR fragments that were functionalized with multiple biotin and digoxigenin groups, respectively. The 7.9-kbp DNA is the ligation product of residues 755–1153 of the pbluescrIISK plasmid (Stratagene), residues 4214–6163 and 9023–11,220 of lambda- phage DNA (Promega) and residues 1945–5304 of the pSFV1 plasmid (Stratagene). The DNA tethers were attached to streptavidin-coated 1.0-µm-diameter MyOne or 2.8-μm-diameter M270 superparamagnetic beads (Invitrogen) by incubation in PBS (Sigma).

### 4.3 RNA constructs

Measurements on RNA (Fig. 3) used a 4218 bp fully double-stranded RNA construct with multiple biotin labels at one end and multiple digoxigenin labels at the other end that enable attachment to streptavidin-coated magnetic beads and the anti-dig- coated flow surface, respectively. RNA constructs were generated using a two-step in vitro transcription procedure described in Ref. [6].

### 4.4 Angle measurements in the magnetic torque tweezers

Torque measurements in magnetic tweezers are based on determination of the rotation angle *θ* of the tethered bead and Eq. 1. Two basic strategies are available for tracking of the rotation angle. The first approach involves direct tracking of *θ* from images and typically (but not always [16]) requires use of an additional fiducial marker bead on the magnetic bead [9], pairs of magnetic beads [17], or a separate rotor bead [18,19]. An alternative strategy is to track the beads’ (x,y,z)-positions and to convert (x,y) to angle, which is the approach used in all data sets presented here except for Ref. [3]. Tracking *θ* from the (x,y)-position exploits the specific tethering geometry of magnetic beads under a predominantly vertically (i.e., along z, Fig. 1) aligned field. In conventional MT, the magnetic field is oriented in the (x,y)-plane and the preferred magnetic axis of the bead (***m_0_***, Fig. 1, middle panel top) aligns with the external field [20]. In the magnetic torque tweezers or freely orbiting tweezers, the magnetic field is vertically aligned and the preferred magnetic axis of the bead aligns vertically (Fig. 1, bottom right). In this magnetic configuration, the center position of the bead traces out a circle [12]. For measurements of the angle *θ*, we first track the bead position during rotation of the magnets and fit a circle to the (x,y)-positions. Using this initial fit, the subsequent positions in (x,y) can be converted to rotation angle *θ,* as described in detail in [4,12].
